# Direct HPV E6/Myc interactions induce histone modifications, Pol II phosphorylation, and hTERT promoter activation

**DOI:** 10.18632/oncotarget.22036

**Published:** 2017-10-25

**Authors:** Yiyu Zhang, Aleksandra Dakic, Renxiang Chen, Yuhai Dai, Richard Schlegel, Xuefeng Liu

**Affiliations:** ^1^ Department of Pathology, Center for Cell Reprogramming, Georgetown University Medical Center, Washington, DC 20057, USA

**Keywords:** telomerase, papillomaviruses, oncoproteins, histones, RNA polymerase II

## Abstract

Human Papillomavirus Viruses (HPVs) are associated with the majority of human cervical and anal cancers and 10-30% of head and neck squamous carcinomas. E6 oncoprotein from high risk HPVs interacts with the p53 tumor suppressor protein to facilitate its degradation and increases telomerase activity for extending the life span of host cells. We published previously that the Myc cellular transcription factor associates with the high-risk HPV E6 protein *in vivo* and participates in the transactivation of the hTERT promoter. In the present study, we further analyzed the role of E6 and the Myc-Max-Mad network in regulating the hTERT promoter. We confirmed that E6 and Myc interact independently and that Max can also form a complex with E6. However, the E6/Max complex is observed only in the presence of Myc, suggesting that E6 associates with Myc/Max dimers. Consistent with the hypothesis that Myc is required for E6 induction of the hTERT promoter, Myc antagonists (Mad or Mnt) significantly blocked E6-mediated transactivation of the hTERT promoter. Analysis of Myc mutants demonstrated that both the transactivation domain and HLH domain of Myc protein were required for binding E6 and for the consequent transactivation of the hTERT promoter, by either Myc or E6. We also showed that E6 increased phosphorylation of Pol II on the hTERT promoter and induced epigenetic histone modifications of the hTERT promoter. More important, knockdown of Myc expression dramatically decreased engagement of acetyl-histones and Pol II at the hTERT promoter in E6-expressing cells. Thus, E6/Myc interaction triggers the transactivation of the hTERT promoter by modulating both histone modifications, Pol II phosphorylation and promoter engagement, suggesting a novel mechanism for telomerase activation and a new target for HPV- associated human cancer.

## INTRODUCTION

The E6 oncoprotein of a high risk human papillomavirus type 16 (HPV16) has been shown to activate telomerase activity in epithelial cell types predominantly by inducing transcription of the hTERT (human telomerase reverse transcriptase) gene [1–6]. By itself, E6 can immortalize a subpopulation of human mammary epithelial cells [7–9], and E6 in cooperation with E7 can immortalize primary human foreskin keratinocytes (HFKs) [10, 11]. Interestingly, both hTERT and Myc can substitute for E6 in E6/E7-mediated immortalization of primary HFKs [7, 12], indicating that telomerase activation constitutes a major immortalizing activity of E6. In previous studies, we and others have shown that E6-mediated hTERT transactivation is not p53 degradation-dependent or PDZ motif-dependent [3, 7, 12–14], and requires E6-AP [15] and Myc cooperation [4, 16–18]. We have shown that E6 and Myc associate *in vivo* and both bind to the hTERT promoter in primary HFKs [2, 19], and in that way, Myc determines E6-responsiveness of the hTERT promoter [20].

Myc regulates the expression of up to 10-15% of the cellular genes [21] controlling metabolic processes, macromolecular synthesis, the cell cycle and apoptosis [22]. hTERT, the catalytic subunit of telomerase, is one of the Myc targets [23]. Upregulation of hTERT transcription and the consequent increase of telomerase activity is a critical event during the course of cellular immortalization and malignant transformation [24, 25]. For example, Myc is a direct activator of hTERT in human breast and fibroblasts cells [12, 23, 26, 27]. Overexpression of Myc in these cells increases telomerase, thereby immortalizing these types of cells [27, 28]. Overexpressed Myc also induces hTERT in HFKs [2, 12, 23, 29], while this is not sufficient to immortalize HFKs [7]. Interestingly, endogenous Myc is too weak to activate telomerase in HFKs, since we and others have shown that endogenous Myc binds to hTERT promoter without activating transcription in primary HFKs [19].

In the current study, we evaluated the interaction of E6 with the Myc-Max-Mad network and its effect on the hTERT activation. Our data conclusively demonstrated that E6/Myc interactions trigger the transactivation of the hTERT promoter by modulating both histone modifications and Pol II phosphorylation, which highlight the complexity of E6 interactions with cell regulatory proteins.

## RESULTS AND DISCUSSION

### HPV E6 associates with Myc *in vivo* and *in vitro*

**Figure 1:**
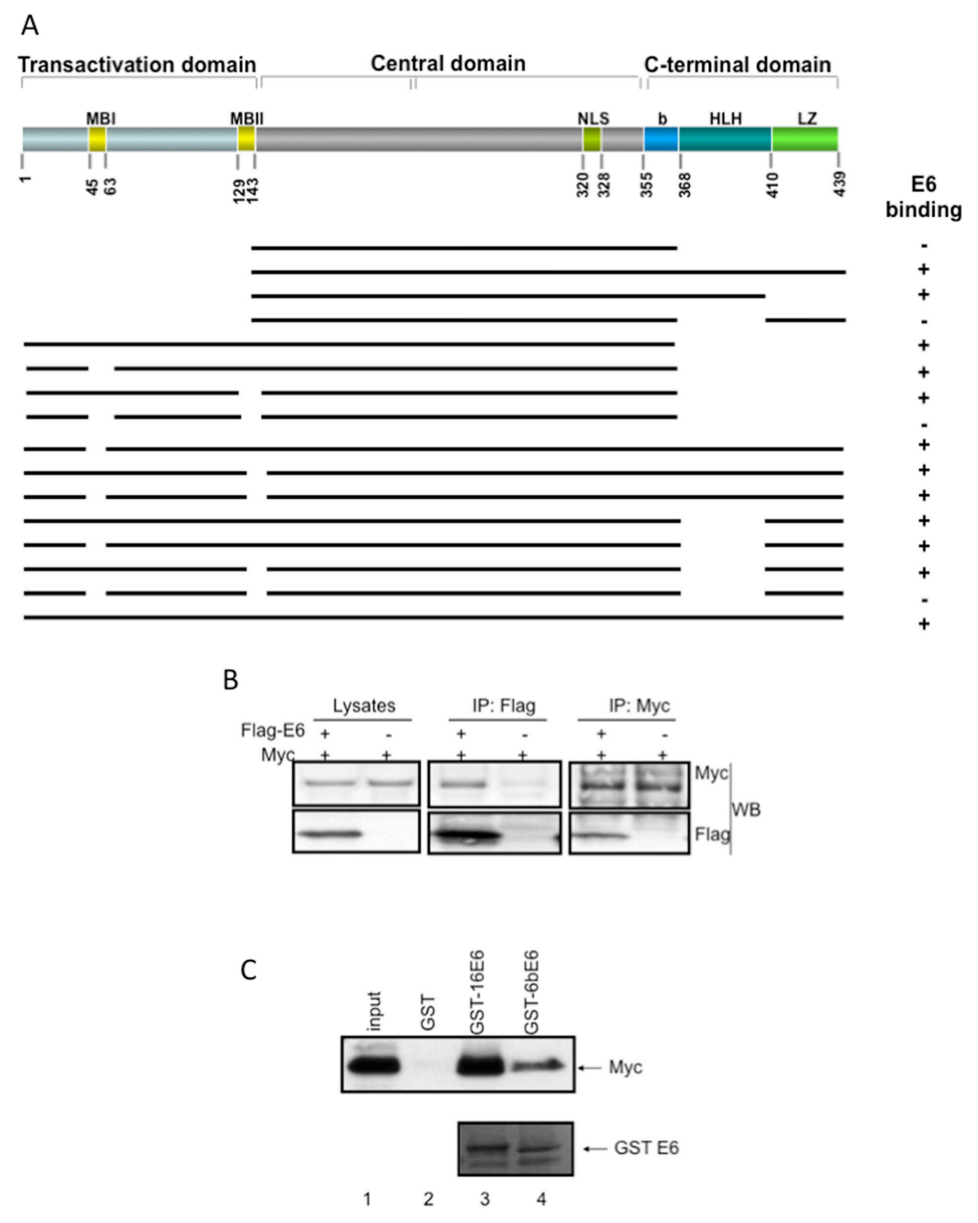
HPV E6 associates with Myc *in vivo* and *in vitro* **(A)** Diagram of Myc structure and schematic representation of the deletion constructs of Myc. MBI: Myc box I; MBII: Myc box II; MBIII: Myc box III; NLS: nuclear localization signal; b: basic; HLH: helix-loop-helix domain; LZ: leucine zipper. **(B)** HPV E6 interacts with Myc *in vivo*. Extracts of 293T cells transfected with pcDNA-Myc and/or pCMV3xFlag-E6 as indicated with “+” and “-”, respectively, were immunoprecipitated with the Flag antibody (central) or Myc antibody (N-262, Santa Cruz) and blotted with Myc (N-262, up) and Flag (M2, down) antibodies. Lysates were also analyzed with straight WB, as shown in left. **(C)** HPV E6s interact with Myc *in vitro*. The wt Myc was made by IVT, and the same amount of the IVT proteins were subjected to GST alone, GST-16E6 or GST-6BE6 pulldown assays. The captured products were separated on 4-20% SDS-PAGE and blotted with monoclonal anti-Myc antibody (9E10). The lower panel indicates the equal amount of GST-E6 products. The low risk HPV E6 (6BE6) has a relative lower affinity to associate with Myc compared to high risk E6 (16E6).

We have previously shown that AU1-tagged HPV E6 associates with Myc oncoprotein in COS cells and primary human foreskin keratinocytes (HFKs) [19]. Our results showed that AU1 antibody immunoprecipitated Myc protein, however, we were not able to show that Myc antibody immunoprecipitated E6 in those cells. In this study, we used Flag-E6, an epitope-tagged E6 protein construct, and co-transfected it with Myc into 293 cells. We used both anti-Flag and anti-Myc antibodies to verify protein expression (Figure 1B, left panel) and to detect E6/Myc association by co-immunoprecipitation (Co-IP). Our results confirmed that in 293 cells E6 co-immunoprecipitates with Myc *in vivo* in both directions (Figure 1B). More important, we also observed the association of E6 and Myc in HFK cells that were treated with proteasome inhibitor, MG-132, for 4hrs before cell lysis (data not shown). To further test whether E6 directly associated with Myc, we performed GST pull-down experiments *in vitro* with GST-E6 fusion protein and *in vitro* translated Myc. Consistent with the above results, GST-E6, but not GST alone, strongly bound to Myc (Figure 1C, upper panel, lane 1-3). In addition, we also compared the ability of high- and low-risk HPV E6 to bind to Myc *in vitro*. Our data showed that 6BE6 (low risk) had a much lower ability to interact with Myc compared to the 16E6 (high risk) (Figure 1C, upper lane 4), suggesting that the ability of E6 to associate with Myc might be important for its transforming function.

### Both transactivation and HLH domains of Myc are required for E6/Myc interaction

To dissect the domains (Figure 1A) of Myc interacting with E6, we first generated a Myc expression vector containing the central domain fragment (aa 143-368), as shown in Figure 1A and Supplementary Figure 1A. Since it was difficult for us to find an appropriate antibody recognizing all of our constructs, we labeled *in vitro* translated (IVT) Myc fragments with ε-labeled biotinylated lysine-tRNA complex (Promega) and performed a GST pull-down experiment. The IVT proteins that were associated with GST-E6 fusion protein were visualized with Transcend™ Non-Radioactive Translation Detection Systems (Promega). This labeled central domain of Myc did not associate with either GST or GST-E6 proteins (Supplementary Figure 1A, upper panel, lane 1, 2, 3). After we generated two larger Myc fragments to include the C-terminus, GST-E6 clearly interacted with these two fragments, aa 143-439 (Supplementary Figure 1A, upper lane 4-6) and aa 143-410 (Supplementary Figure 1A, upper lane 7-9). Interestingly, E6 did not interact with a Myc fragment aa 143-439 containing a deletion of HLH domain (Supplementary Figure 1A, upper lane 10-12). Thus, we conclude that HLH domain of Myc fragment with the central domain and C-terminus is critical for E6 binding. When we extended the central domain to include the Myc N-terminus (aa 1-368), E6 could bind Myc (Supplementary Figure 1A, lower panel, lane 1-3), including the fragments with a deletion of either MBI (Supplementary Figure 1A, lower panel, lane 4-6) or MBII (Supplementary Figure 1A lower panel, lane 7-9) separately. However, E6 did not interact with an Myc fragment containing deletions of both MBI and MBII (Supplementary Figure 1A lower panel, lane 10-12). Therefore, these data suggest that the discontinuous Myc HLH and MBI and MBII domains participate in E6 binding.

To confirm whether these domains are required for the association of full length Myc with E6, we generated additional Myc mutants with single, double and triple deletions of the full length Myc. All of these mutant Myc proteins were recognized by monoclonal Myc antibody, 9E10. We used this Myc antibody to detect Myc protein by western blot and the verification of GST pulldown of IVT Myc proteins. As shown in Figure 1A and Supplementary Figure 1B, E6 associated with wild type Myc, mutant Myc proteins with individual deletion of either MBI, or MBII, or HLH domains (Supplementary Figure 1B, upper panel, lane 1-12), as well as Myc mutants with double deletion of domains derived from MBI, MBII, and HLH (Supplementary Figure 1B, lower panel, lane 1-9). However, E6 failed to associate with Myc mutant that had triple deletion of MBI, MBII and HLH (Figure 1A and Supplementary Figure 1B. 1F, lower panel, lane 10-12). Thus, these data further confirm that MBI, MBII, and HLH are three Myc domains critical for association with E6.

### E6 interacts with Myc/Max complex, not Max alone

The above data demonstrate that E6 interacts with Myc *in vitro* and *in vivo*, and we showed previously that both E6 and Myc bound the hTERT promoter in primary keratinocytes [19]. Since Myc protein is part of a more complex and dynamic protein network, we hypothesized that E6 might also interact with other Myc-associated proteins such as Max and thereby modulate hTERT transcription. To evaluate this possibility, we made both wild type Myc and Max proteins by *in vitro* translation, and performed GST pull down experiments with IVT Myc alone, Max alone and premixed Myc/Max. As described above, GST-E6 interacted with wt Myc (Supplementary Figure 2, upper panel, lane 1-3), but not Max protein (Supplementary Figure 2, lower panel, lane 4 and 5). However, when Max was premixed with Myc, E6 was able to pull down Myc (Supplementary Figure 2, upper panel, lane 6, even though a weaker signal), as well as Max (Supplementary Figure 2, lower panel, lane 6). These data suggest that E6 interacts with a Myc/Max complex, but not Max alone.

### E6 does not change the occupancy of Myc/Max proteins at the hTERT promoter

We have previously shown that both E6 and Myc proteins associate with the the endogenous hTERT promoter in primary keratinocytes [19]. Interestingly, Myc binds to the hTERT promoter in keratinocytes independently of E6. We and others did not observe significant change in Myc bound to hTERT promoter in the presence of E6 by regular ChIP assay [19], although one group suggested that Myc replaces repressive USF bound to the promoter in E6 expressing cells [20]. Although E6 does not alter Myc expression, it is possible that E6 recruits more Myc to the hTERT promoter or stabilizes Myc on the promoter, thereby transactivating hTERT transcription. Since the regular ChIP assay is not a highly quantitative method and might not allow us to observe a critical difference in Myc bound the promoter, we performed a quantitative chromatin immunoprecipitation (Q-ChIP) assay. The data demonstrated that both Myc and Max occupied the hTERT promoter in an empty vector- and in E6- expressing cells, and that there was no significant change in the amount of Myc or Max bound to the promoter in both these two types of cells (Supplementary Figure 5). This indicates that E6 modifies Myc or the Myc/Max complex *via* direct interaction at the hTERT promoter without changing the amount of the proteins bound to the promoter.

### E6 does not alter Myc expression, but increases Myc phosphorylation

We and others have shown previously that E6 does not induce Myc expression in HFKs [19]. In this study, our data showed that E6 did not alter mRNA levels of Myc, Max or Mad in HFKs (Supplementary Figure 3). E6 did not alter Myc protein levels either (Supplementary Figure 3). p53 protein was blotted as a positive control for E6 expression and function (Supplementary Figure 3).

**Figure 2:**
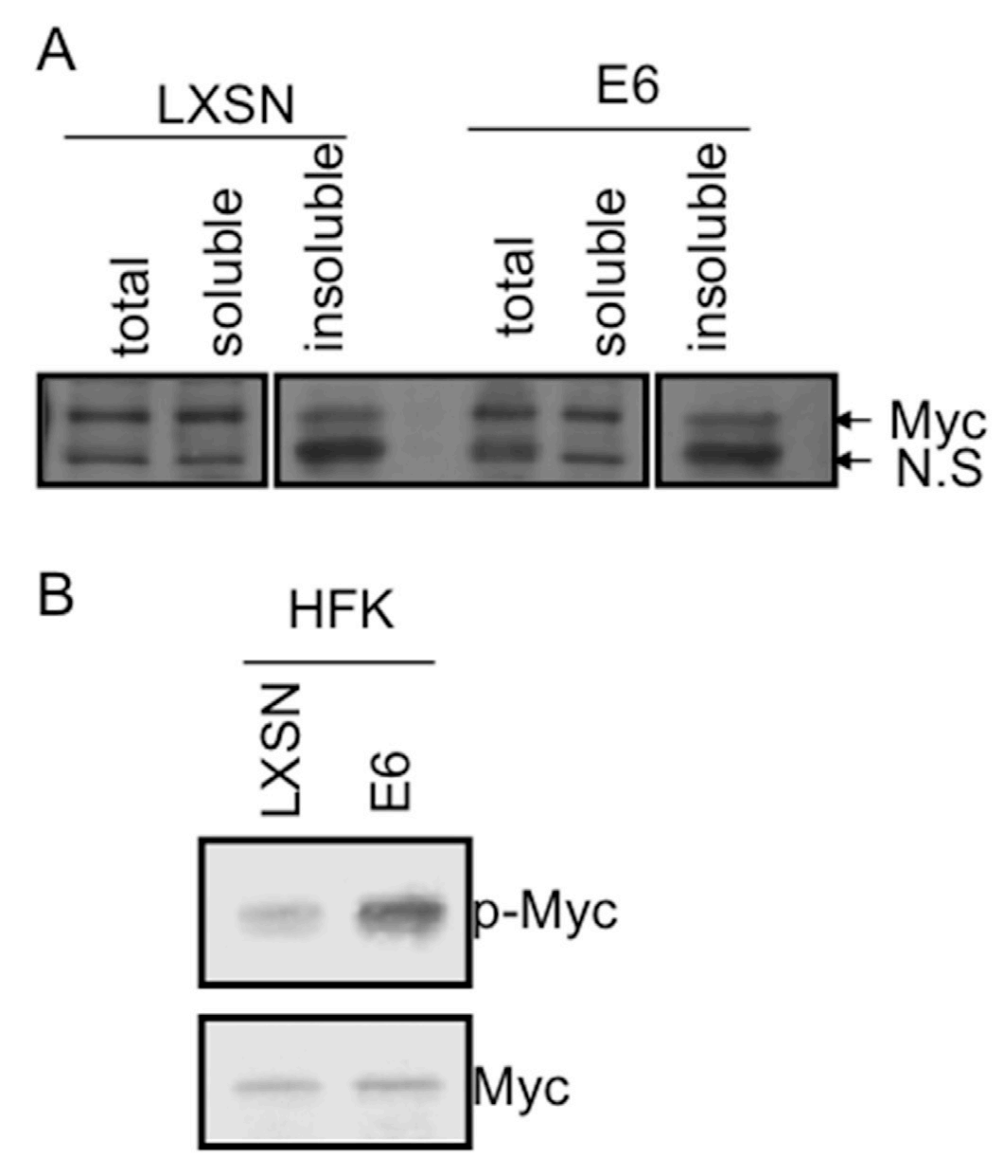
E6 increases Myc phosphorylation without induction of Myc expression **(A)** Levels of total, soluble and unsoluble Myc proteins in keratinocytes expressing either LXSN or E6. RIPA lysates from keratinocytes expressing LXSN or E6 were centrifuged at 14,000 rpm at 4°C. RIPA lysates (total, indicated as T), supernatants (soluble, indicated as S1), pellets (unsoluble, chromatin-associated, indicated as S2) were blotted with 9E10 monoclonal Myc antibody. NS indicates non-specific band. E6 does not alter levels of total, soluble, or unsoluble Myc in keratinocytes. **(B)** E6 induces Myc phosphorylation. Western blot analysis for endogenous phosphorylated c-Myc proteins in pLXSN- and E6-transduced HFK cells. Immunoblot analysis was performed on whole cell lysates using Thr58/Ser62 phospho-c-Myc-specific antibody. The protein blot was then stripped and reprobed for total c-Myc to demonstrate equal expression and loading.

While E6 does not alter the total levels of cellular Myc protein, it is possible that E6 induces changes in the stability of a small subpopulation of Myc in the cell (i.e. the DNA-associated or hTERT promoter-associated forms). This would not have been detected in earlier studies of mass Myc abundance. The concept that there are different pools of Myc protein was recently illustrated by Tworkowski et al. [31] who have shown that, while Myc is generally a very unstable protein (t ½ = 20 min), there is a small percentage (10% or less) which has a much longer half life (t ½ = 120 min) and is highly-ubiquitinated and resistant to extraction by RIPA buffer. We postulate that E6/E6AP might force more Myc into this stable active form. We therefore examined the levels of total, soluble and insoluble Myc in HFKs with either LXSN or E6. As shown in Figure 2A, we did not observe a difference in Myc levels in any Myc fraction regardless of the presence of E6.

Finally, we looked at phosphorylated Myc in LXSN or E6 expressing HFKs with antibody against phosphorylated Myc at either T58 alone or T58/S62. Surprisingly, we found that that although E6 did not alter total Myc protein abundance, it significantly increased the level of phosphorylated Myc protein (Figure 2B). The mechanism responsible for this alteration in phosphorylation is being explored.

### Dissociation of Myc/Max complexes with a small molecule (10058-F4) dramatically blocks E6-induced hTERT promoter activity

**Figure 3:**
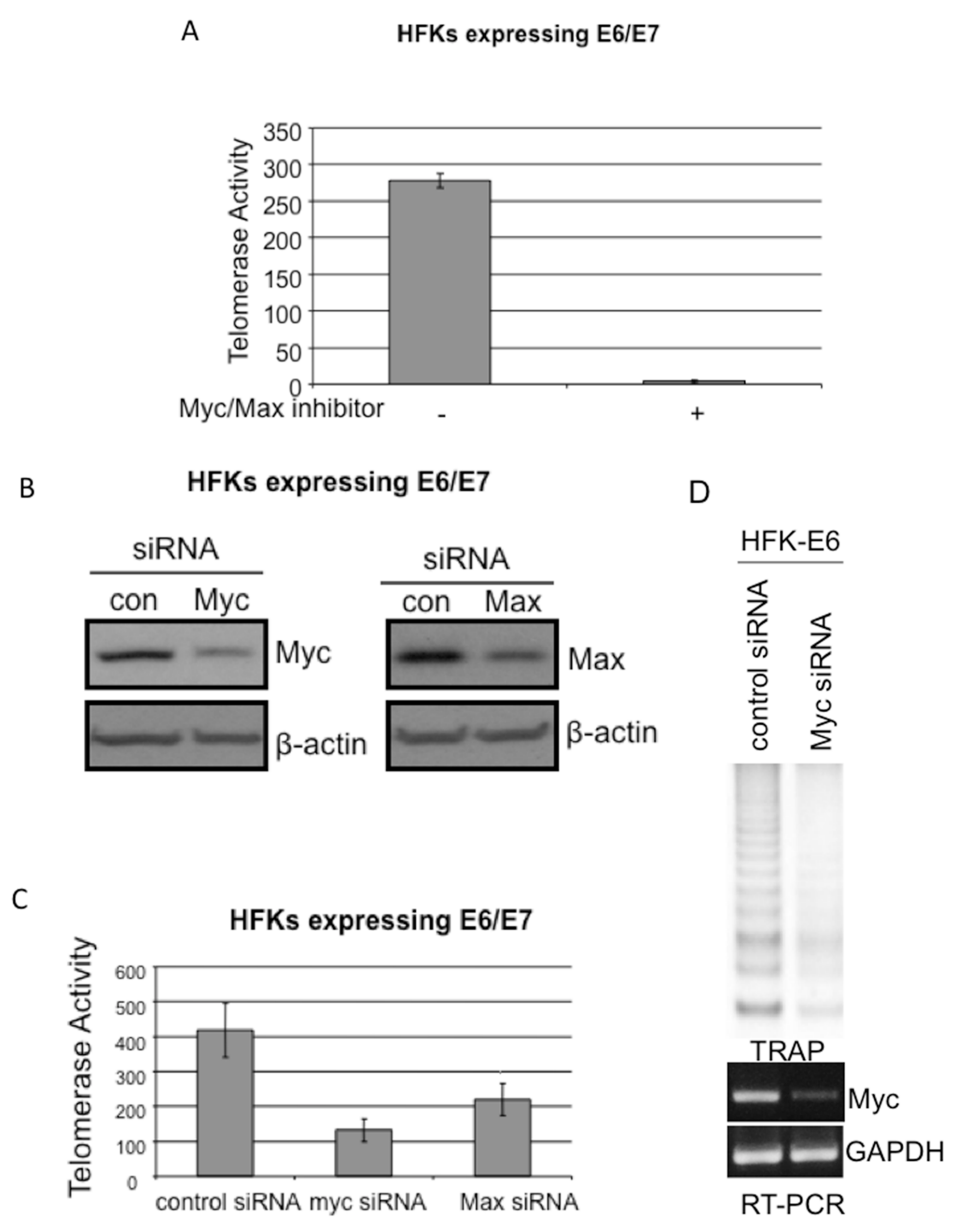
Knockdown of either Myc or Max decreases telomerase activity in E6E7 immortalized cells or tumor-derived cell lines **(A)** Dissociation of Myc/Max complex blocks dramatically telomerase in E6/E7 immortalized cells. The HFKs expressing E6/E7 were treated with 4 nM of 10058-F4 for 16 hrs. Cell lysates were subjected to quantitative real time TRAP assay (QRT-TRAP) and a significant decrease in telomerase activity after 10058-F4 treatment was observed compared to that in control cells. **(B)** siRNAs decrease expression of Myc and Max proteins. Cells were transfected with either control siRNA (24 and 48 hours), or Myc specific (24 hours), or Max specific (48 hours) siRNA duplexes in 6-well plates and cell lysates were used for Western blot. Beta actin was blotted as internal controls. **(C)** siRNAs against Myc or Max decrease telomerase activity. The above same siRNA treated cells were lysated with TRAP buffer and QRT- TRAP assay was performed as described in Materials and Methods. Decreased telomerase activities in HFKs expressing E6/E7 were observed after treatment with either Myc or Max specific siRNA duplex. **(D)** Myc is required for E6-induced telomerase activity in HFKs. Myc specific siRNA duplex sufficiently knockdowns Myc expression as shown in lower panel with RT-PCR and significantly decreases E6-induced telomerase activity (upper panel) with regular TRAP assays.

Our previous studies have shown that Myc is important for E6 induced hTERT promoter activity [19]. Since the dimerization of Myc and Max is required for Myc transcription functions [32], we examined whether dissociation of the Myc/Max complex could inhibit E6 activation of the hTERT promoter. We used a commercially available small molecule (10058-F4) which is sufficient to disrupt Myc/Max complexes and inhibit Myc binding to E box elements [33]. The treatment of E6E7 expressing cells with 10058-F4 dramatically blocked telomerase activity, as shown in Figure 3A. These results suggested that dissociation of the Myc network proteins, without overexpression or knockdown of any of them, effectively affected E6-mediated hTERT transcription.

### Knockdown of Myc abrogates E6 mediated transactivation of the hTERT promoter

As we have shown previously, overexpression of Myc induced the hTERT promoter activity 2-3 fold higher than vector control, and enhanced E6 induction of the hTERT promoter activity [19]. To further confirm the requirement of Myc for E6 transactivation of hTERT promoter, we questioned whether knockdown of endogenous Myc might inhibit E6 mediated hTERT promoter activity. We co-transfected Myc specific siRNA duplexes (Damarcon, Smartpool) together with hTERT reporter and E6 expression vector into primary keratinocytes. Myc siRNA was sufficient to decrease E6 mediated hTERT promoter activity, compared to scrambled duplexes [14]. We found that knockdown of Myc expression with siRNA led to a significant decrease (~70%) in telomerase activity compared to control siRNA in E6E7 expressing cells (Figure 3B and 3C), and a similar inhibition of telomerase activity in E6 expressing cells (Figure 3D). We next transfected hTERT promoter reporter gene with either control or Myc specific siRNA into HeLa cells, SiHa cells or E6E7 expressing keratinocytes (Data not shown). hTERT promoter activity was reduced 70-80% with Myc siRNA treatment compared to that with control siRNA. These data confirmed that endogenous Myc is required for E6 activation of telomerase, in either E6, E6E7 expressing cells or HPV positive tumor cells. Thus, although previous results suggested that E6 does not alter Myc expression, Myc expression is required for E6 transactivation of hTERT promoter in primary keratinocytes.

### Myc antagonists, Mad and Mnt, inhibit E6 induced hTERT activity

**Figure 4:**
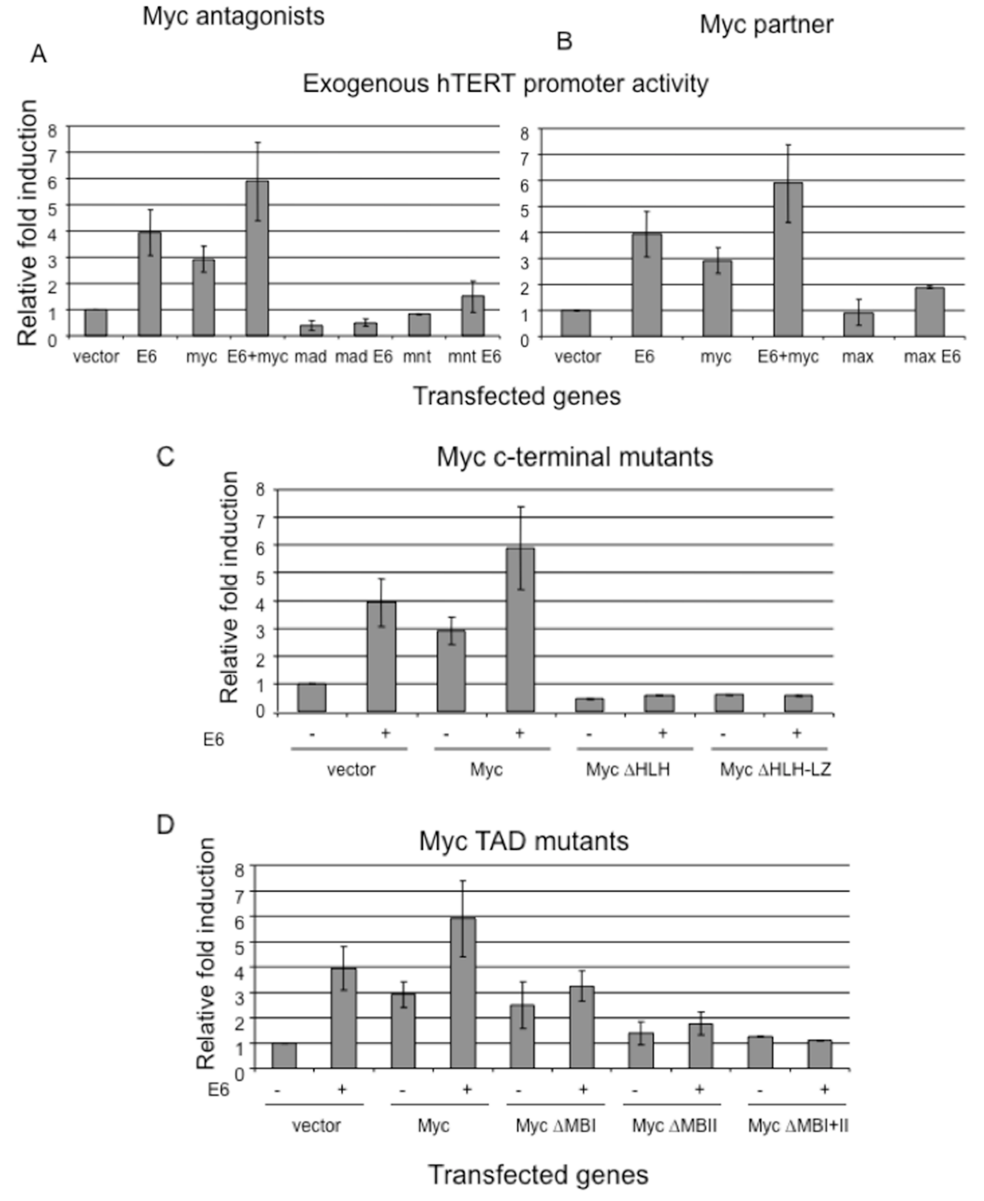
Interference with the balance of Myc/Max network abolishes E6 induction of hTERT promoter Primary HFKs were transfected with the hTERT core promoter and either wt Myc or its mutants, or together with E6. The pRL-CMV *Renilla reniformis* reporter plasmid was also transfected into the cells to standardize for transfection efficiency. Luciferase activity was measured 24 hours after transfection using the Dual luciferase reporter assay system (Promega). Relative fold activation reflects the normalized luciferase activity induced by Myc or its mutants with or without E6 compared to the normalized activity of vector control. The value of pGL3B-empty vector activity was set to 1. Error bars show the standard deviation from at least three independent experiments. **(A)** Myc antagonists, Mad and Mnt, block E6 induced hTERT promoter. **(B)** Myc partner, Max, reduces E6-mediated hTERT promoter activity. **(C)** C-terminal of Myc is required for E6-mediated hTERT promoter activity. Myc mutants missing C-terminal, which is responsible for dimerization with Max and binding to DNA, inhibit E6 induction of hTERT promoter. **(D)** Myc transactiviation domain (MBII, not MBI) is critical for E6-mediated hTERT promoter activity. Myc mutant missing MBII, not MBI, decreases E6 induction of the hTERT promoter.

In general, Myc, Max, and Mad proteins form a network that regulates gene expression, proliferation, apoptosis and differentiation. Transcription-competent Myc/Max heterodimers are the active form of Myc [34]. Max also heterodimerizes with the other bHLH-Zip proteins, such as Mad1, Mxi-1 (Mad2), Mad3, Mad4, and Mnt [35]. These alternative dimers bind the E-Box and actively repress transcription, and therefore antagonize both the transcriptional and transforming activities of Myc [36]. We hypothesized that these Myc antagonists might block E6 mediated hTERT transcription through a competitive dimerization with Myc partner, Max. Therefore, we cotransfected primary keratinocytes with the hTERT reporter and two Myc antagonists, Mad1 and Mnt, as well as with an E6 expression vector. As anticipated, overexpression of either Mad1 or Mnt efficiently blocked E6 induced hTERT promoter activity (Figure 4A). Consistent with other reports [37, 38], overexpression of Myc rescued Mad or Mnt repression of hTERT promoter. These data suggested that the suppression of E6-dependent hTERT promoter by Mad or Mnt was carried out through dimerization with Max and antagonizing Myc function.

### Both overexpression and knockdown of Myc partner, Max, inhibits E6 induction of hTERT

Max is the obligatory DNA-binding and dimerization partner for all the members of the Myc/Max/Mad network which recognize E-box sequence of target genes. Max is the only member of network that efficiently homodimerizes and binds to E box element *in vitro* [39, 40]. However, Max lacks a transcription regulatory domain [40]. Our above data have shown that change of expression level of either Myc or its antagonists regulate E6 mediated hTERT promoter activity. In order to test whether change in Max expression can also affect E6 induced hTERT transcription, we cotransfected Max expression vector and hTERT promoter with either vector or E6. Overexpression of Max, unlike overexpression of Mad1, did not change the relative activity of uninduced hTERT promoter, while it significantly inhibited E6 induced hTERT promoter activity (Figure 4B). More importantly, we sufficiently inhibited expression of endogenous Max in E6E7 expressing cells or HPV positive cancer cell lines (SiHa and HeLa cells) with siRNA duplex treatment for 48 hrs. Although Max has a relative longer half life in excess of 24h compared to other network proteins [41]. This inhibition led to decrease telomerase activity by 30-40% (Figure 3B and 3C). The lower percentage of inhibition compared to Myc siRNA possibly reflects a relative longer half life of Max over Myc. However, we obtained a similar inhibition of hTERT promoter activity as Myc when Max siRNA was cotransfected to the above cells together with hTERT reporter. Therefore, we concluded that overexpression or knockdown of Max, a central protein of the Myc network, sufficiently inhibited E6 mediated hTERT promoter activity.

### HLH-LZ domain of Myc is critical for E6-induced hTERT promoter activity

The C-terminus of Myc contains a basic-helix-loop-helix-leucine-zipper (bHLH-LZ) domain that mediates dimerization, which is a prerequisite for DNA binding through the adjacent basic (b) motif. Dimerization with Max and DNA binding are essential for the oncogenic, mitogenic, and apoptotic functions of Myc [42]. We speculated that the C-terminus of Myc might be required for induction of the hTERT promoter by Myc itself or by E6.

Again, we performed luciferase assays for the hTERT promoter using HLH-LZ deletion Myc mutant alone, or together with E6. Results clearly demonstrated that this Myc mutant completely lost its ability to induce the hTERT promoter (Figure 4C). Compared to wt Myc, the relative fold induction of the promoter was even lower than that with vector control. More important, this mutant also blocked E6-mediated hTERT promoter activity. Altogether, our data strongly suggest that the ability of Myc to dimerize with Max and to bind to DNA is not only essential for the function of Myc itself, but also it is critical for E6 transactivation of hTERT.

### Transactivation domains, especially the MBII domain of Myc, are required for Myc and E6 dependent hTERT transactivation

As shown in Figure 1A, the N-terminus of Myc contains a functional transcriptional activation domain (TAD) with two highly conserved motifs, Myc boxes (MB) I and II [43]. TAD is found to be necessary to co-operate with H-ras to induce transformation of primary rat embryo fibroblasts [44]. These domains are also necessary for Myc to induce transformation, apoptosis and block differentiation [45–48], although two other Myc homology domains have been characterized recently [49]. The MBII domain is critical for Myc transactivation of target genes *via* recruiting transcriptional cofactors, such as TRAAP [50, 51]. We hypothesized that this domain was also essential for transactivation of hTERT by Myc or E6. To test the hypothesis, we generated Myc mutants which were missing either MBI, or MBII, or both. We co-transfected the hTERT reporter with either E6, or Myc/Myc mutants, or E6 plus Myc/Myc mutants. As expected, E6 expression alone induced hTERT promoter activity 3-5 fold higher than the vector control (Figure 4D). Myc alone led to a 2-3 fold induction, while expression of both E6 and Myc caused 5-8 fold higher induction (Figure 4D). Interestingly, the deletion of MBI did not affect the promoter activity by itself, compared to the wild type Myc (Figure 4D), but this mutant did further increase induction when cotransfected together with E6. Strikingly, the Myc mutants with deletion of either MBII or both MBI and MBII showed greatly reduced hTERT promoter induction compared to the wt Myc. The relative fold induction of promoter by these mutants was similar to vector control. More importantly, these mutants also abolished E6-mediated hTERT promoter activity, which indicated that TAD of Myc, especially MBII, is not only required for Myc-induced hTERT promoter activity, but also for E6 transactivation of the hTERT promoter. To insure that the results with the Myc mutant proteins were not simply due to altered cell localization, we examined their intracellular localization by immunofluresence and confirmed that all Myc mutants were present in the nucleus similar to wt Myc. (Supplementary Figure 4).

### E6 induces epigenetic changes at the hTERT promoter

**Figure 5:**
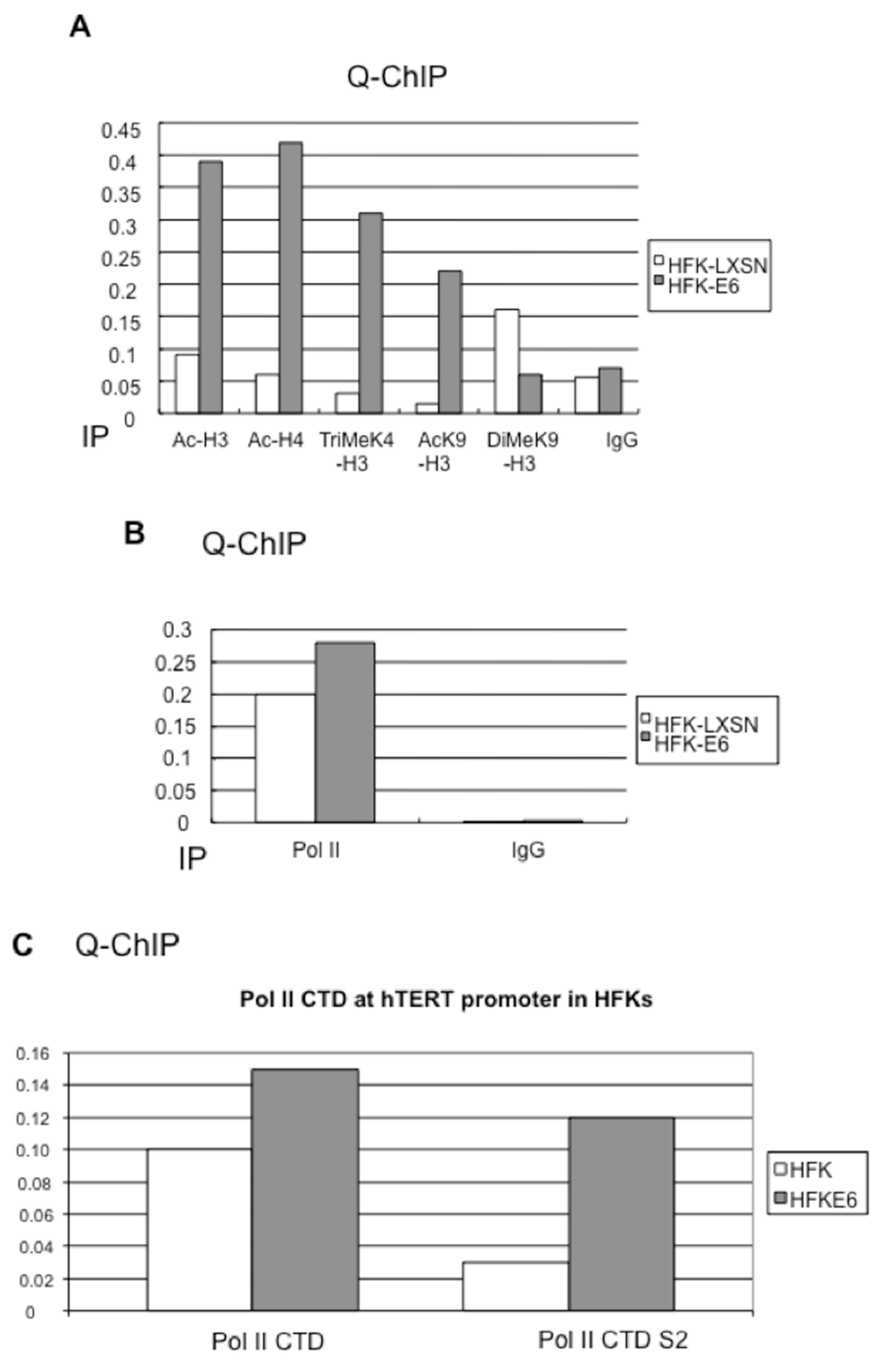
HPV16 E6 induces chromatin changes and phosphorylation of the RNA Polymerase II (Pol II) CTD at the hTERT promoter **(A)** E6 induces chromatin changes at the hTERT promoter. ChIP lysates from HFK-LXSN and HFK-E6 cells were precipitated with antibodies against Ac-H3, Ac-H4, Tri-Me K4-H3, Di-Me-K9 H3, or Ac-K9 H3, and mouse and rabbit IgG mixture were also included as a negative control. E6 significantly increases histone acetylation (Ac-H3, Ac-H4) at the hTERT promoter. The transcription permissive histones (Tri-Me-K4 H3 and Ac-K9 H3) are increased in E6 expressing cells compared to HFK with vector. However, a transcriptional repressive histone DiMeK9-H3 is slightly decreased in E6 expressing cells. **(B)** RNA Polymerase II is preloaded at the hTERT promoter prior to its activation. Antibody against RNA Pol II and IgG were used for IP and precipitates were subjected to real time PCR. Pol II binds to the hTERT promoter in HFK before its activation and E6 does not significantly alter Pol II bound to the hTERT promoter. **(C)** E6 induces Pol II phosphorylation at serine 2 (S2) of CTD. Antibodies against Pol II CTD and S2 phosphorylated CTD were used for quantitative ChIP assays. Upon expression of E6, Pol II protein is increased only slightly (1.5 fold) on the hTERT promoter. More significantly, the Pol II protein shows a 5-fold increase in phosphorylation of its CTD serine 2 residue.

Histones, due to their structural role of chromatin packacging, have a critical function in regulating gene transcription, and histone modifications such as acetylation and methylation of H3 and H4 are associated with active gene transcription. To determine if E6-induced telomerase activity is associated with histone modification, we screened several modified histones at the hTERT promoter using ChIP assays. Our results showed that E6 significantly increased Ac-H3, Ac-H4, Tri-Me-K4 H3, and Ac-K9 H3 at the hTERT promoter (Figure 5A), while a transcription repressive modified histone, Di-Me-K9 H3 was slightly decreased at the promoter. Thus, E6 induced epigenetic modifications at the hTERT promoter which correlated positively with telomerase activation. This is in agreement with previous studies showing that E6 affects hTERT and increases telomerase activity through chromatin structure modifications of the hTERT promoter, such as histone acetylation and demethylation [52, 53].

### RNA polymerase II is preloaded on the “silent” hTERT promoter and E6 induces S2 phosphorylation of Pol II CTD

The typical RNA polymerase II (Pol II) transcription cycle begins with the binding of specific activators upstream of the core promoter (including the TATA box and transcription starting site). This event leads to and recruitment of the adaptor complexes such as SAGA or mediator, both of which in turn facilitate binding of the general transcription factors (GTFs). Pol II together with TFIID, TFIIA, and TFIIB factors form the preinitiation complex [54, 55]. In human mesenchymal stem cells, a significant level of paused Pol II is present at the repressed inactive hTERT promoter [56]. Trichostatin A (TSA), an HDAC inhibitor, overcame hTERT repression and increased in promoter-specific histone acetylation. It was hypothesized that chromatin changes could remove this pause and that activation of the hTERT promoter requires adjacent chromatin modifications [56].

Initially we speculated that E6-induced induction of the hTERT promoter might be due to increased recruitment of Pol II to the promoter. To evaluate this, we performed Q-ChIP assays with anti-Pol II antibody, as well as IgG as a negative control. Interestingly, our data also demonstrated that Pol II complexes were bound to the hTERT promoter regardless of transcriptional activity (Figure 5B). We also obtained similar results when we used antibody against Pol II CTD (Figure 5C, left). Thus, E6 appears to activate the hTERT promoter *via* a post-Pol II recruitment mechanism. Productive elongation is thought to be regulated by the cyclin-dependent kinase P-TEFb (positive transcription elongation factor), which has been shown to phosphorylate the C-terminal domain (CTD) of Pol II [57, 58]. Others have previously shown that promoters such as fos, Myc, and HSP70 are also bound by stalled, or paused, RNA polymerases [55]. Indeed, it appears that Myc may play a role in transcription elongation through recruitment of P-TEFb and phosphorylation of Pol II CTD [59].

The most significant finding was that E6 induced a 5-fold increase in the phosphorylation of Pol II on CTD serine 2 (Figure 5C, right). Phosphorylation at this site is highly correlated with the productive elongation of gene transcripts [60–62]. Thus, similar to Myc, we found that Pol II engages the hTERT promoter even in the quiescent state and that E6 expression only marginally increases this association. However, we found that promoter-resident Pol II was differentially phosphorylated during E6 activation. This suggests that E6 does not recruit Pol II to the hTERT promoter, but rather mediates changes in its phosphorylation. These data provide a new potential mechanism for E6 to regulate cellular gene transcription.

### Myc is required for E6-mediated epigenetic changes at the hTERT promoter

**Figure 6:**
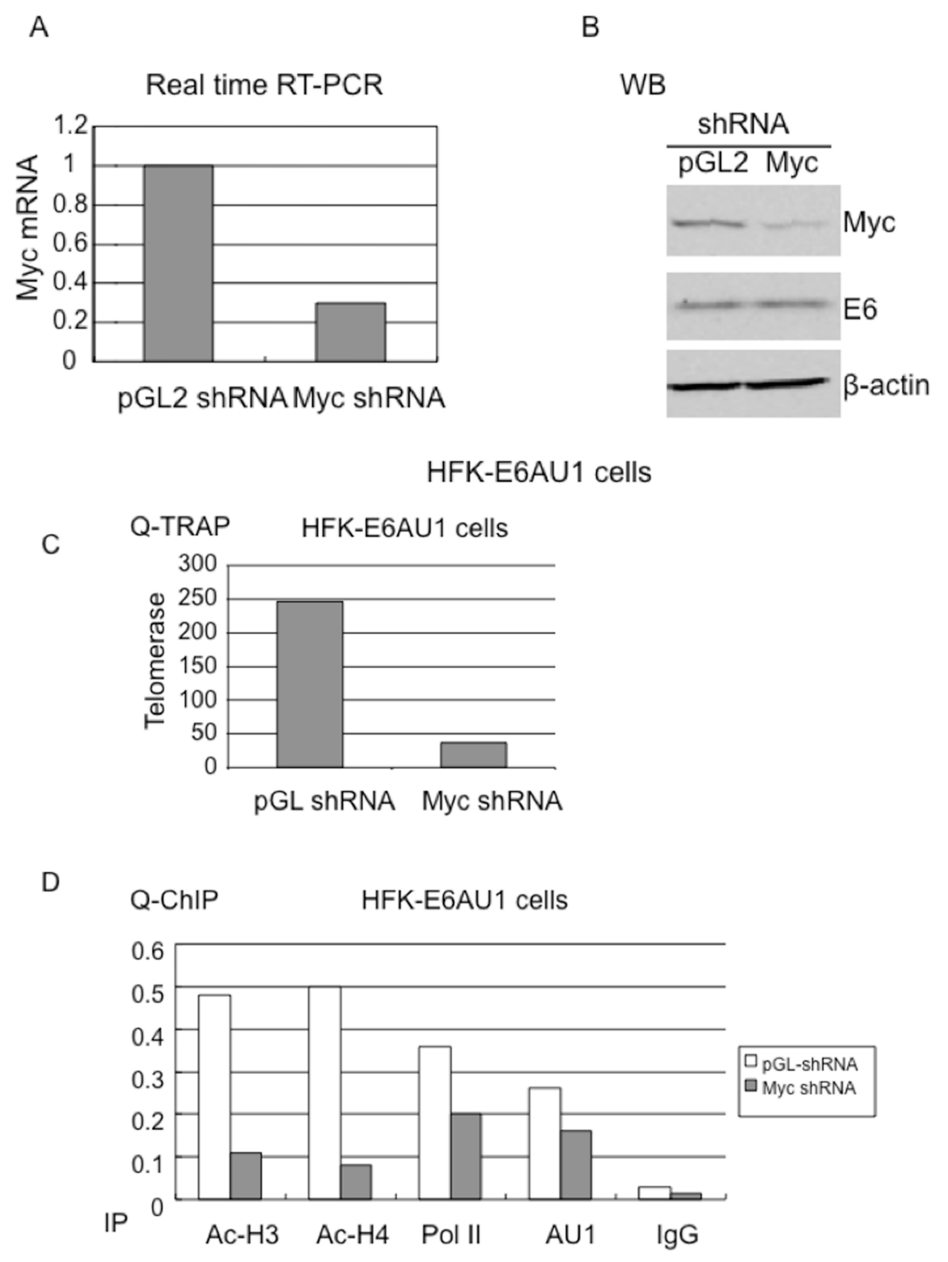
Myc is required for E6-induced chromatin changes at the hTERT promoter HFK-E6AU1 cells were infected with retroviruses expressing pGL2 shRNA or Myc shRNA and selected with puromycine for 4-5 days. **(A)** shRNA mediated knockdown of Myc expression. Total cellular RNA was isolated with Trizol (Invitrogen) and QRT-PCR was performed with Myc specific primers. GAPDH mRNA was used to normalize Myc expression. **(B)** E6 expression does not change in Myc shRNA expressing cells. The above cells were lysated with 2x SDS buffer and subjected to Western blot with antibodies against Myc, AU1 or beta-actin. Myc is decreased in Myc shRNA expressing cells and E6 expression remains identical in both pGL2 and Myc shRNA expressing cells. **(C)** Myc shRNA decreases telomerase activity in E6 expressing cells. The above same shRNA treated cells were lysated with TRAP buffer and QRT-TRAP assay was performed as described in Materials and Methods. A decreased telomerase activity in HFKs expressing E6AU1 were observed after treatment with Myc shRNA, not pGL2 shRNA. **(D)** Myc is required for E6 induced chromatin changes at the hTERT promoter. HFKs expressing E6AU1 or empty vector, LXSN, were lysated with ChIP buffer and precipitated with antibodies against Ac-H3, Ac-H4, AU1, or Pol II. Mouse and rabbit IgG mixture were also included as a negative control. Myc shRNA significantly descreases histone acetylation (Ac-H3, Ac-H4) and slightly decreases E6 and Pol II at the hTERT promoter in E6 expressing cells.

Myc protein is associated with the hTERT promoter and, although E6 does not increase this association, we wanted to determine whether Myc was required for E6-induced epigenetic changes at the hTERT promoter. We transduced HFK cells expressing the E6-AU1 protein with retroviruses expressing either control pGL2-shRNA or pGL2-Myc shRNA [63]. Real time RT-PCR and WB demonstrated a significant knockdown of Myc expression (Figure 6A and 6B) with Myc shRNA compared to the control, pGL2 shRNA. The shRNA did not affect E6 expression, as verified by AU1 antibody blotting (middle of Figure 6B). Corresponding to the knockdown of Myc, we observed a significant inhibition of telomerase activity (Figures 3D and 6C). In addition, we found a significant decrease in acetylated histones at the hTERT promoter compared to control shRNA (Figure 6D). Therefore, we conclude that E6-induced epigenetic changes at the hTERT promoter are Myc dependent. More important, our ChIP data demonstrated that Myc knockdown decreased association of E6 and Pol II with the hTERT promoter (Figure 6D). This further confirmed our previous study that Myc determines responsiveness of the hTERT promoter to E6 [13, 20].

## SUMMARY

**Figure 7:**
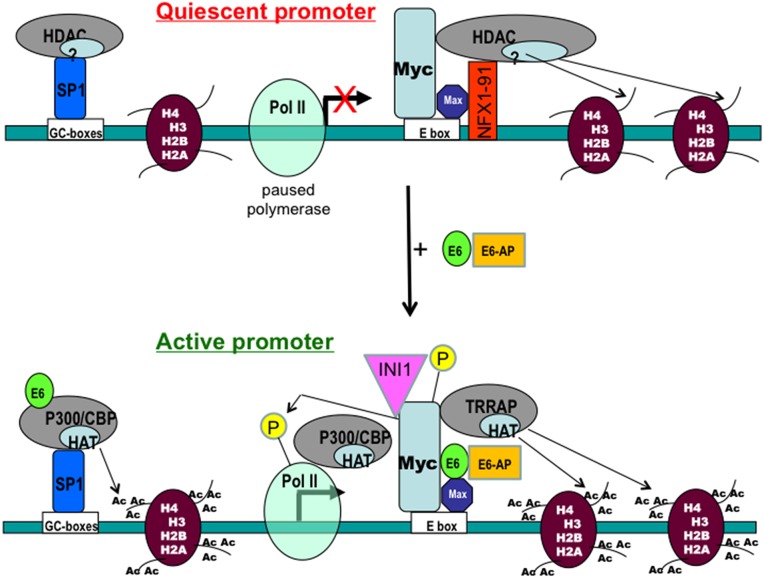
A working model for regulation of the hTERT promoter by E6 and Myc proteins Results from the current and previous studies are summarized to illustrate the possible mechanism for regulation of the hTERT promoter by the HPV E6 and Myc oncoproteins. Myc and RNA polymerase II are pre-existing on the “silent” hTERT promoter. E6 induced epigenetic changes may “push” the “paused Pol II complex” on the promoter, leading an active transcription.

We confirmed previous published data that E6 and Myc associate *in vitro* and *in vivo* [2, 19]. Then we demonstrated that both the transactivation domain and HLH domain of Myc were required for E6/Myc association and these domains were required for transactivation of the hTERT promoter, by either Myc or E6. Interestingly, E6 bound to a Myc/Max complex. While ectopic Myc activated the hTERT promoter, overexpression of the Myc antagonists, Mad or Mnt, significantly blocked E6-mediated transactivation of the hTERT. Overexpression of exogenous Max or knockdown of endogenous Max with siRNA decreased hTERT promoter transactivation. Previous studies have indicated that Myc is physically associated with both the active and silent hTERT promoter. Similar to Myc, our ChIP assays suggested that RNA polymerase II (Pol II) was also preloaded on the hTERT promoter even in the quiescent state [30], and became phosphorylated following E6 expression. In addition, E6 induced epigenetic histone modifications of the hTERT promoter. Most importantly, since knockdown of Myc expression dramatically decreased engagement of acetyl-histones and Pol II at the hTERT promoter in E6-expressing cells, these results indicate an important role for the E6/Myc interaction in the epigenetic modification of the hTERT promoter and its transactivation. It is very possible that Myc bound to the hTERT promoter is responsible for pre-recruitment of Pol II, since both are bound to the promoter regardless of transcriptional activity. Thus, altogether our results indicate that E6/Myc interaction might activate the hTERT transcription via epigenetic changes and histone modifications (especially phosphorylation of Myc and Pol II CTD) at the “silent” hTERT promoter (Figure 7).

## MATERIALS AND METHODS

### Plasmids

p3XFlag-E6, GST-E6, pJS55 vector, pJS55-16E6, pJSS55-16E7, pLXSN vector and pLXSN-16E6, pLXSN-16E7, pLXSN-16E6E7, pGL2 shRNA, pGL2 shRNA Myc, pGL3-basic (pGL3B) and pGL3B-hTERT core promoter (defined as pGL3B-255 previously) were as described previously [19], pcDNA-Myc, pcDNA-Mad, pcDNA-Max constructs were the gifts from Drs. I. Horikawa and J.C. Barrett (National Cancer Institute, National Institutes of Health, Bethesda), pcDNA-Myc, pcDNA-MycΔMBI, pcDNA-MycΔMBII, pcDNA-MycΔMBI+II were gifts from Dr. Lars-Gunnar Larsson (Karolinska Institut, Stockholm, Sweden). To construct Myc143-368, Myc143-410, Myc143-439, PCR was done with wt Myc as template and primers 5’-CGGGATCCATGCTCGTCTCAGAGAAGCTG-3’ with a BamHI site at 5’ end and 5’-CGGAATTCTTAGTTCCTCCTCTGGCGCTC-3’, 5’-CGGAATTCTTACTCCTCTGCTTGGACGGACAG-3’, and 5’-CGGAATTCTTACGCACAAGAGTTCCGTAG-3’, respectively, with EcoR I sites at 5’ ends. To clone Myc1-368, 1-368ΔMBI, 1-368ΔMBII, 1-368ΔMBI+II, PCR was performed with primers 5’-CGGGATCCATG CCCCTCAACGTTAGC-3’ with a BamH I site at 5’ end and 5’-CGGAATTCTTAGTT CCTCCTCTGGCGCTC-3’ with an EcoR I site at 5’ end using wt Myc, MycΔMBI, MycΔMBII and Myc ΔMBI+II as templates, respectively. The above PCR products were digested with BamH I and EcoR I, then ligated with the BamH I and EcoR I fragment of pcDNA3 (Invitrogen). To construct MycΔ370-409 (or MycΔHLH), an overlapping PCR was used to delete aa370-409 (HLH domain) in wt Myc. Briefly, using wt Myc as DNA template, the first round PCR was done with Myc 5’ end primer 5’-CGGGATCCATG CCCCTCAACGTTAGC-3’ with a BamH I site and deletion region primer 5’-GAGCTTTTGCTCCTCGTTCCTCCTCTGGCG-3’, and Myc 3’ end primer 5’-CGGAATTCTTACGCACAAGAGTTCCGTAG-3’ with an EcoR I site and another deletion region primer 5’-CGCCAGAGGAGGAACGAGGAGCAAAAGCTC-3’, respectively. Then, using a mixture of the first PCR products as template, the second round PCR was done with the above Myc 5’ end and 3’ end primers. The second PCR product was digested with BamH I and EcoR I, and then inserted into the BamH I and EcoR I fragment of pcDNA3. The same strategy and primers were used for construction of MycΔMBI+ΔHLH, MycΔMBII+ΔHLH, and Myc ΔMBI+II+ΔHLH using MycΔMBI, MycΔMBII, and Myc ΔMBI+II instead of wt Myc as templates.

### Glutathione S-transferase (GST) pull-down assays

GST-E6 and GST proteins were expressed in *Escherichia coli* induced with IPTG (Sigma I-6758), and conjugated to Glutathione-Agarose beads (Sigma G-4510). The expression of GST-E6 and GST protein was confirmed by SDS-PAGE and Coomassie Blue staining. Myc, Myc mutants and Max proteins were translated *in vitro* with TNT^®^ T7 Quick Coupled Transcription/Translation System (Promega L1170). Myc fragments (Myc1-368, 143-368, 143-410, 143-439, 143-368+410-439, 1-368ΔMBI, 1-368ΔMBII, 1-368ΔMBI-II) were biotinylated during *in vitro* translation. The same amount of the IVT proteins were subjected to GST alone or GST-E6 pulldown assays. After electrophoresis with 4-20% of SDS-PAGE, the proteins were transferred to PVDF membranes and visualized with Transcend™ Chemiluminescent Non-Radioactive Translation Detection System (Promega, L5080) for Myc fragments and with blotting with Myc monoclaonal antibody (9E10) for wt Myc and its full-length based mutants (with deletions of either individual or combinations of MBI, MBII, HLH domains).

### Retroviruses

Retrovirus packaging cells, SD3443, were transfected with pLXSN, vectors with either E6 or E7, or both E6 and E7 as described above using LipofectAmine 2000 (Invitrogen) as suggested by the manufacturer. Culture supernatants containing retrovirus were collected 24 hours after transfections.

### Cell culture and stable lines selection

Primary human foreskin keratinocytes (HFKs) were cultured from neonatal foreskins from Georgetown University Hospital as described [64] and maintained in keratinocyte growth media (Gibco-BRL), supplemented with gentamycin (50 μg/ml). Establishment of stable cell lines was described as previously [65]. HeLa and SiHa cells were maintained and cultured in complete DMEM with 10% FBS in our laboratory at Georgetown University.

### Immunoprecipitation and western blot

pcDNA-Myc, and p3XFlag-E6 or their vector control were co-transfected to 293T cells through Calcium phosphate transfection. Cells were lysed with RIPA buffer (50 mM Tris-HCL, pH 7.4, 150 mM NaCL, 1% NP-40, 0.5% Sodium deoxycholat, 0.1% SDS and fresh PMSF, protease inhibitor cocktail) after 24 hrs of transfection. 500 μg of lysates were subjected to immunoprecipitation with either anti-Myc antibody (N-262, Santa Cruz, SC-764) or Anti-FLAG M2 Affinity Gel (Sigma, A-2220). The immunoprecipitates were subjected to SDS-PAGE and blotted with Anti-Myc antibody (9E10) for upper part and Anti-FLAG M2 monoclonal antibody for lower part, respectively.

### Myc/Max inhibitor

Myc/Max inhibitor, 10058-F4 [(*Z,E*)-5-(4-Ethylbenzylidine)-2-thioxothiazolidin-4-one], was purchased from CalBiochem Inc. (Cat ^#^475956). DMSO was used to desolve 10058-F4, 4 nM was used to treat primary HFKs with transfection for Luciferase assays or E6E7 tranduced HFK for 16 hrs. Then, cells were harvested for luciferase or telomerase measurement.

### Real-time quantitative RT-PCR (QRT-PCR)

Real time quantitative RT-PCR for detection of Myc mRNA and GAPDH was performed as described previously [1, 4, 14].

Real-time QRT-PCR was performed on the Bio-Rad iCycler MyiQ for quantitation of Myc mRNA using primers (5’-ACCACCAGCAGCGACTCTGA-3’ and 5- TCCAGC AGAAGGTGATCCAGACT-3’) and β-actin was also amplified with primers (5’-GCTTGCTGATCCACA TCTGC -3’ and 5’-TGGACATCCGCAAAGACCTG-3’) as an internal control.

### Quantitative chromatin immunoprecipitation (Q-ChIP) assay

HFKs transduced with either pLXSN or HPV E6 were grown to 80-90% confluency in a 100-mm dishes. ChIP was performed as described previously [PMID: 12821782]. Normal rabbit IgG (Santa Cruz), rabbit anti-Myc polyclonal antibody (N-262, Santa Cruz), Max, Mad, Pol II, Pol II CTD, Pol II CTD S2 were applied to appropriate immunoprecipitation. DNA from IPs as well as from input samples was subjected to a real time PCR with hTERT promoter specific primers (5’-GAGCTGGAA GGTGAAGGGGC-3’ and 5’-TTCCCACGTGCGCAGC AGGA-3’). The relative occupy was normalized by input DNA.

### Immunofluorescence microscopy

Cells were grown on 22 × 22 mm glass cover slips in six-well cluster plates, fixed in 4% (w/v) paraformaldehyde at room temperature for 20 minutes. Cells were permeabilized by 0.1% saponin (10 min) and blocked with.10% donkey serum in PBS (Invitrogen) for 20 minutes at room temperature. Myc antibody (N-262, Santa Cruz) and Alexa Fluor-conjugated donkey secondary antibodies (Molecular Probes) were used to stain Myc expression. DNA was stained by treatment for 5 minutes with 10 uM Hoechst dye 33342 (Sigma).

### siRNAs and shRNAs

The siRNA target sequences were as follows: for c-myc siRNA duplexes targeting four different region in myc (smartpool from Dharmacon), the targets are nt 437-455 5’-CAGAGAAGCTGGCCTCCTA-3’, nt 360-378 5’-CGACGAGACCTTCATCAAA-3, nt 1263-1281 5’-GAAACGACGAGAACAGTTG-3’ and nt 908-926 5’-CCACACATCAGCACAACTA-3’. 60-70% confluency of HeLa cells or 70-80% confluency of SiHa cells, pLXSN-16E6E7 transduced HFKs were tranfected with a final concentration of 40 nM of negative control siRNA, myc specific siRNA duplex mixture (Dharmacon, Smartpool) or Max specific siRNA with LipofectAmine 2000 (Invitrogen) following the manufacturer's instructions. Cells were harvested for Luciferase assay 24 hrs posttransfection, and for TRAP assay 48 hrs posttransfection, respectively. For Luciferase assay, SiHa, HeLa, pLXSN-16E6E7 transduced HFK cells were co-tranfected with siRNA duplexes plus pGL3B-hTERT and *Renilla reniformis* luciferase gene as described below. shRNA against Myc and pGL3 were gifts from Dr. Ernest Martinez [63]. Retroviruses expressing shRNA were made as described above and infect E6-AU1 expressing HFK.

### Luciferase assay

Luciferase assays were performed as described previously [6, 19].

### Real-time quantitative telomeric repeat amplification protocol (Q-TRAP)

Real time quantitative TRAP for detection of telomerase activity was performed as described previously [1, 4, 14].

## SUPPLEMENTARY MATERIALS FIGURES AND TABLES



## Abbreviations

HPVs: Human Papillomavirus Viruses
hTERT: human telomerase reverse transcriptase
HFKs: human foreskin keratinocytes
E6-AP: E6 associated proteins
PDZ: post synaptic density protein (PSD95), Drosophila disc large tumor suppressor (Dlg1), and zonula occludens-1 protein (zo-1)
Co-IP: co-immunoprecipitation
GST: Glutathione S-transferases
IVT: *in vitro* translated
HLH: helix-loop-helix
MBI: Myc box I
MBII: Myc box II
ChIP: chromatin immunoprecipitation
bHLH-LZ: basic-helix-loop-helix-leucine-zipper
TAD: transcriptional activation domain
TRAAP: transformation/transcription domain-associated protein
Pol II: RNA polymerase II
CTD: C-terminal domain
GTFs: general transcription factors
TFIID: the transcription Factor II D
TFIIA: the transcription Factor II A
TFIIB: the transcription Factor II B
TSA: Trichostatin A
HDAC: histone deacetylases
Q-ChIP: quantitative chromatin immunoprecipitation
HSP70: heat shock protein 70
P-TEFb: the positive transcription elongation factor
