# Supplementary material for: Direct HPV E6/Myc interactions induce histone modifications, Pol II phosphorylation, and hTERT promoter activation
Source: Oncotarget. 2017 Oct 25;8(56):96323–39. doi: 10.18632/oncotarget.22036 (PMC5707103; doi:10.18632/oncotarget.22036)
Supplement: Supplementary file 1 [file oncotarget-08-96323-s001.pdf]

Direct HPV E6/Myc interactions induce histone modifications, Pol II phosphorylation, and hTERT promoter activation

SUPPLEMENTARY MATERIALS

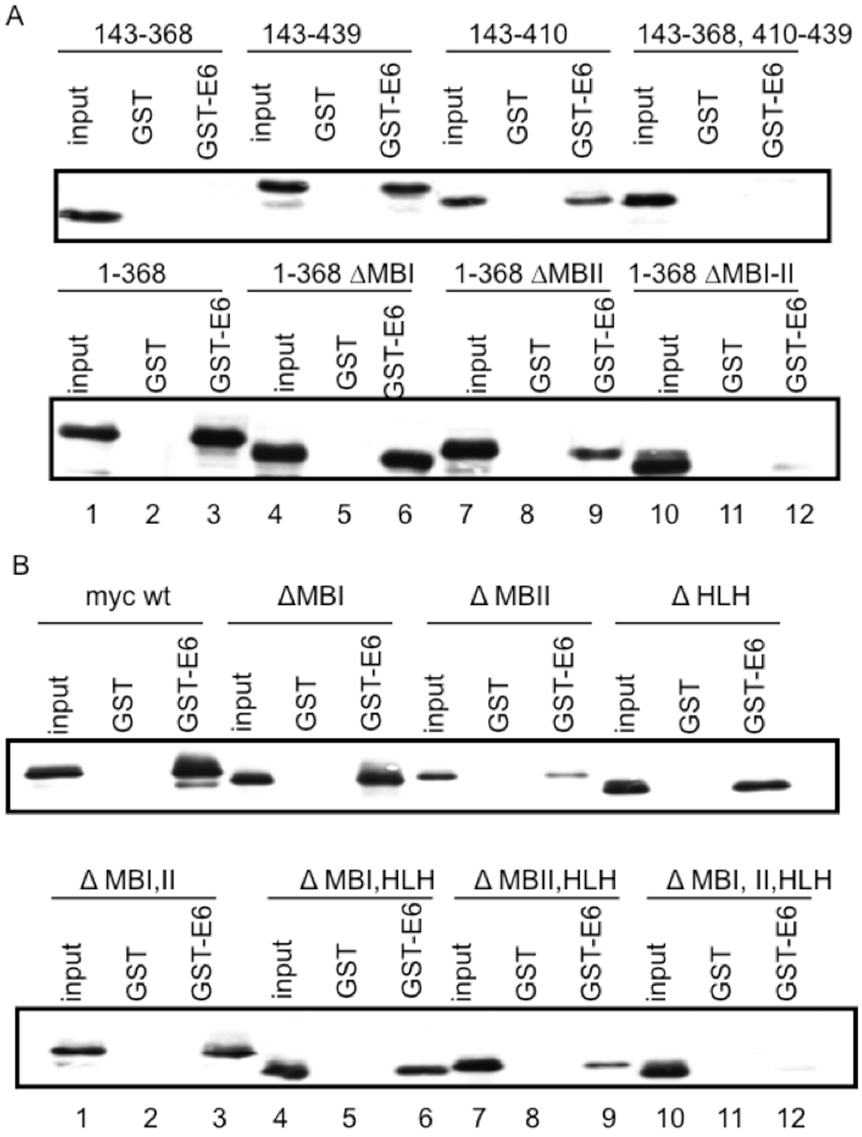

Supplementary Figure 1: (A) Capture of N-, C-termini and central domain of Myc by GST-E6. Myc fragments were translated and biotinylated with TNT *in vitro* transcription and translation system (IVT) (Promega). The lysate lane represents 10% of the IVT protein used in the GST pulldown assays. The same amount of the IVT proteins were subjected to GST alone or GST-E6 pulldown assays. After electrophoresis with 4-20% of SDS-PAGE, the proteins were transferred to PVDF membranes and visualized with Transcend™ Chemiluminescent Non-Radioactive Translation Detection System (Promega, L5080). **(B)** E6/Myc interaction requires MBI, MBII, and HLH domains. The wt Myc and its full-length based mutants (with deletions of either individual or combinations of MBI, MBII, HLH domains) were made by IVT and captured with either GST or GST-E6, captured Myc or its mutants were blotted with monoclonal anti-Myc antibody (9E10).

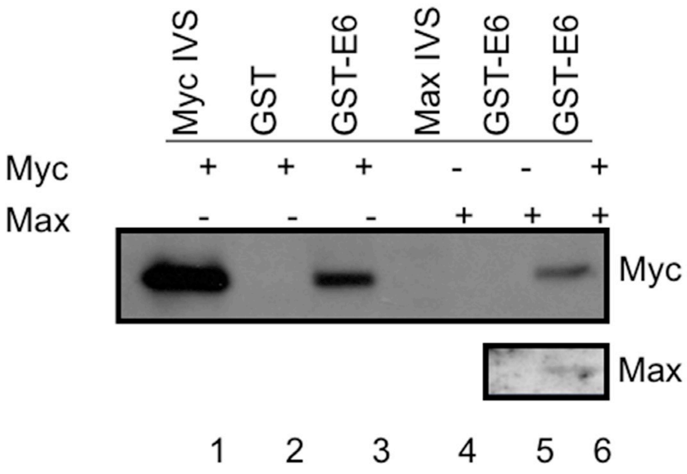

Supplementary Figure 2: HPV E6 interacts with Myc/Max complex, not Max alone. The wt Myc and Max proteins were translated as described in Materials and Methods, the same amount of total lysates with either Myc or Max alone or both Myc and Max was assayed with GST alone or GST-E6 fusion protein. After SDS-PAGE and transfer, the membrane was cut at round molecular weight of 40 KD, the upper and lower parts were blotted with anti-Myc (9E10) and anti-Max (Santa Cruz, C124), respectively. Lane 1 and 4 presents inputs for Myc and Max, respectively. Obviously, E6 interacts with either Myc alone (upper panel, lane 3) or Myc/Max complex (upper and lower panel, lane 6 and 7), but not with Max alone (lower panel, lane 5).

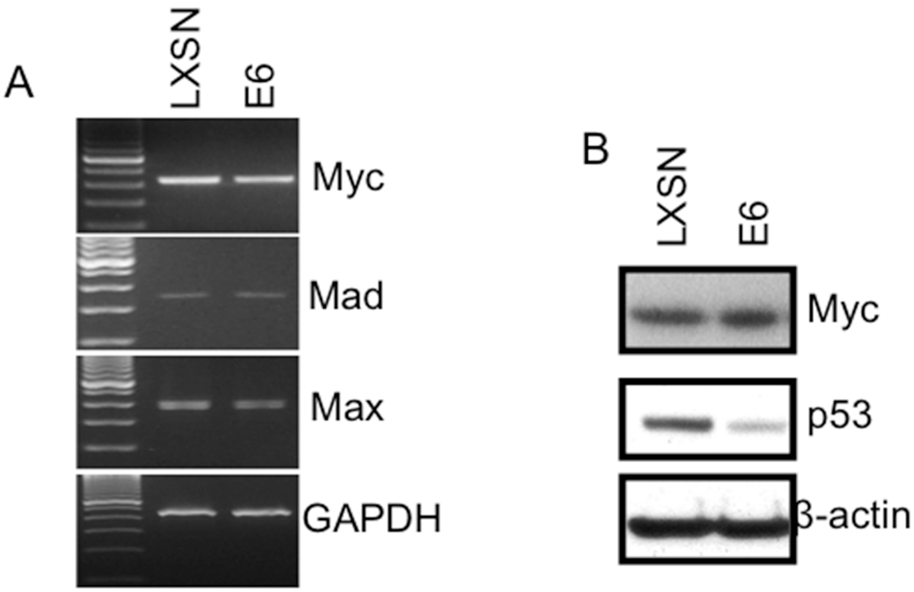

Supplementary Figure 3: E6 does not induce myc expression. **(A)** mRNA expression of myc, max, and mad in keratinocyte lines. Total RNA was isolated from above stable cell lines. The specific sets of primers for myc, max, and mad were used for RT-PCR. A consistent mRNA level of myc, max, mad was detected in all above cell lines. GAPDH mRNA was amplified as an internal control. **(B)** Expression of p53 and Myc proteins in keratinocytes expressing LXSN, and E6. Cell extracts with 2x SDS sample buffer from HFKs expressing empty vector (pLXSN) and E6 were analyzed with anti-p53 (Santa Cruz) and anti-Myc (9E10) antibodies. Blotting with anti-beta actin (Sigma) was used as loading control.

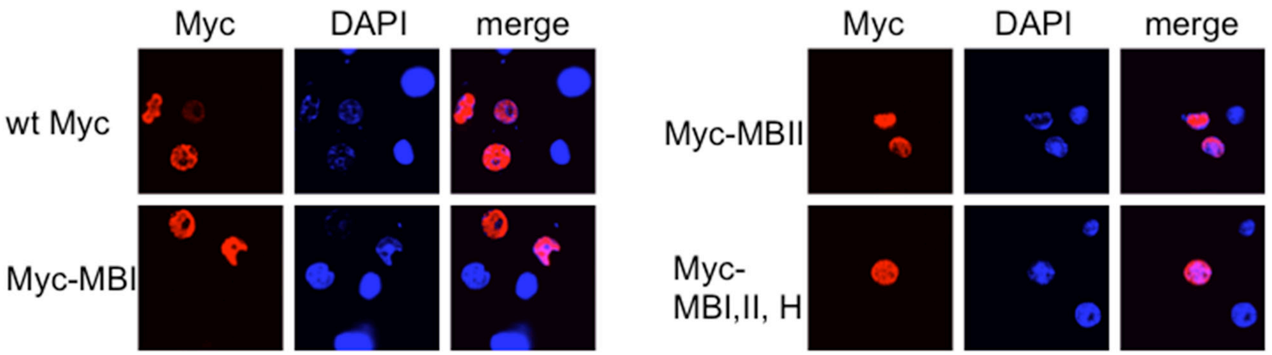

Supplementary Figure 4: Wt Myc and its mutants localize to the nuclei of HFKs. Myc or its mutants constructs were transfected into HFKs and cells were stained with DAPI and Myc antibody (Santa Cruz, N-262).

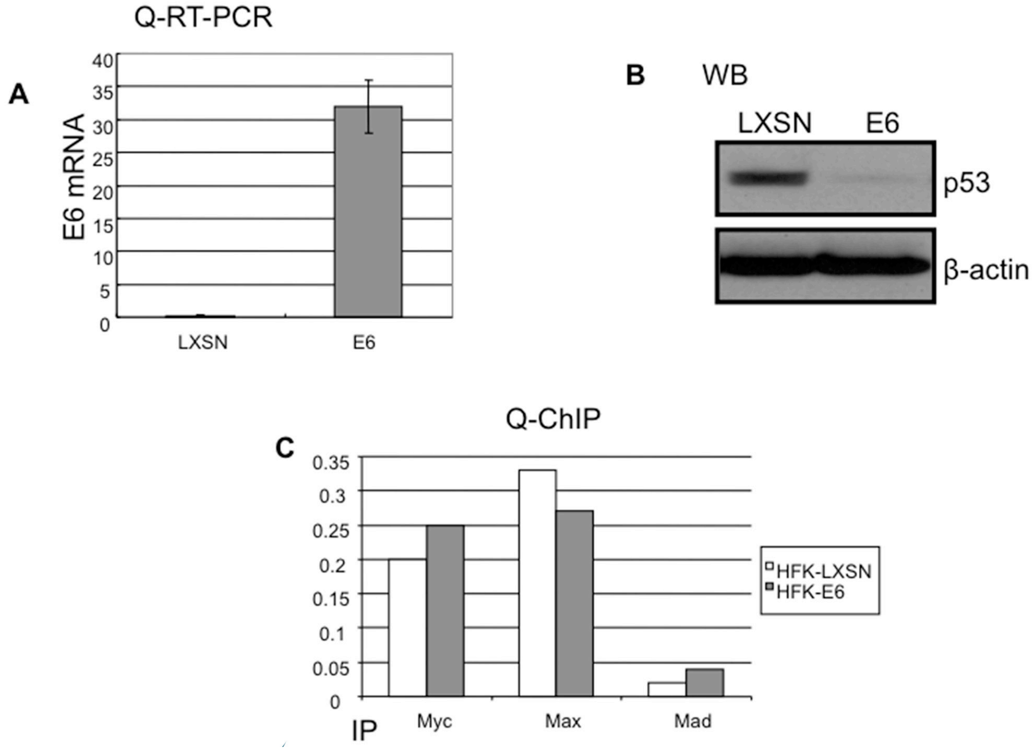

Supplementary Figure 5: HPV16 E6 does not alter Myc abundance at the hTERT promoter. HFKs were infected with retrovirus vector pLXSN or E6, and selected with G418. **(A)** E6 expression. Total cellular RNA was isolated with Trizol (Invitrogen) and the cDNA was synthesized and subjected to real time SYBR Green PCR with E6 specific primers. GAPDH mRNA was used to normalize E6 expression. **(B)** p53 is degraded in E6 expressing cells. The above cells were lysated with 2x SDS buffer and subjected to Western blot with antibodies against p53 or beta-actin. **(C)** E6 does not alter Myc, Max, and Mad bound hTERT promoter in HFKs. A quantitative ChIP assay was done as described in Materials and Methods. DNA from IPs was amplified with SYBR Green kit (Bio-Rad) with hTERT promoter specific primers and normalized as input DNAs (Equivalent to 1% of cell lysate for IP). Both Myc and Max bind to the endogenous hTERT promoter in absence and presence of E6.
